# A Flexible and Transparent PtNP/SWCNT/PET Electrochemical Sensor for Nonenzymatic Detection of Hydrogen Peroxide Released from Living Cells with Real-Time Monitoring Capability

**DOI:** 10.3390/bios13070704

**Published:** 2023-07-03

**Authors:** Da Eun Oh, Chang-Seuk Lee, Tae Wan Kim, Seob Jeon, Tae Hyun Kim

**Affiliations:** 1 Department of Chemistry, Soonchunhyang University, Asan 31538, Republic of Korea; 2 Department of Chemistry, Seoul Woman’s University, Seoul 01797, Republic of Korea; 3 Department of Medical Life Science, Soonchunhyang University, Asan 31538, Republic of Korea; 4 Department of Obstetrics and Gynecology, College of Medicine, Soonchunhyang University Cheonan Hospital, Cheonan 31151, Republic of Korea

**Keywords:** electrochemical sensing, flexible sensor, hydrogen peroxide (H_2_O_2_), platinum nanoparticles, single-walled carbon nanotube (SWCNT) network

## Abstract

We developed a transparent and flexible electrochemical sensor using a platform based on a network of single-walled carbon nanotubes (SWCNTs) for the non-enzymatic detection of hydrogen peroxide (H_2_O_2_) released from living cells. We decorated the SWCNT network on a poly(ethylene terephthalate) (PET) substrate with platinum nanoparticles (PtNPs) using a potentiodynamic method. The PtNP/SWCNT/PET sensor synergized the advantages of a flexible PET substrate, a conducting SWCNT network, and a catalytic PtNP and demonstrated good biocompatibility and flexibility, enabling cell adhesion. The PtNP/SWCNT/PET-based sensor demonstrated enhanced electrocatalytic activity towards H_2_O_2_, as well as excellent selectivity, stability, and reproducibility. The sensor exhibited a wide dynamic range of 500 nM to 1 M, with a low detection limit of 228 nM. Furthermore, the PtNP/SWCNT/PET sensor remained operationally stable, even after bending at various angles (15°, 30°, 60°, and 90°), with no noticeable loss of current signal. These outstanding characteristics enabled the PtNP/SWCNT/PET sensor to be practically applied for the direct culture of HeLa cells and the real-time monitoring of H_2_O_2_ release by the HeLa cells under drug stimulation.

## 1. Introduction

With the rapid development of wearable electronic devices, flexible electrodes are increasingly in demand, since they can conform to different forms and surfaces, such as human skin and other biological tissues [1,2]. Flexible electrodes have potential applications in point-of-care tests and wearable biosensing systems, which allow in situ quantification of various biochemical elements present in the human body, promising personalized health monitoring [3,4,5]. Flexible electrodes provide several benefits over conventional electrodes as supports of biosensing platforms, such as conformability, light weight, portability, low cost, wearability, and facile integration into the transformable system [6,7,8]. To fabricate a high-performance flexible biosensor, the electrode materials should possess chemical or biological functionality, such as catalytic activity, biocompatibility, and chemical stability, along with physical characteristics, such as flexibility and transparency [9,10,11]. Thus, various nanomaterials, including carbon nanomaterials, metal nanowires, conducting polymers, and their composites, have been employed as electrode materials coated on flexible supports (plastic, paper, and textiles) [12,13,14,15,16,17,18,19,20]. We previously reported transparent flexible electrodes based on a single-walled carbon nanotube (SWCNT) network using a poly(ethylene terephthalate) (PET) film substrate for the construction of flexible electrochemical biosensors [21,22]. We used electrochemical approaches, in particular, to prepare the electrochemically doped SWCNT film electrode or dendrimer-grafted SWCNT film electrode, which demonstrated catalytic activity in chemical and biological reactions without the loss of flexibility and transparency. 

In order to broaden the scope of our research on the development of facile SWCNT-based flexible sensing platforms, herein, we demonstrate in situ monitoring of hydrogen peroxide (H_2_O_2_) released from living cells using a functionalized SWCNT/PET film electrode. As a momentous part of reactive oxygen species (ROS), H_2_O_2_ is generated by cellular metabolism and serves as a signaling molecule that modulates a variety of biological processes in the human body, including cell migration, proliferation, and differentiation under physiological conditions [23,24]. However, in cellular environments, overgeneration of H_2_O_2_ can disrupt cellular redox homeostasis, leading to destructive oxidative stress, since its long lifetime allows it to penetrate other cellular compartments and to be accumulated [25]. Consequently, an abnormal level of H_2_O_2_ causes various pathological events, such as cancer, heart attack, aging, Parkinson’s disease, and Alzheimer’s disease [26,27,28,29]. Therefore, the selective and quantitative detection of cellular H_2_O_2_ and monitoring of its dynamic release process from living cells can not only help to elucidate its roles in cellular physiology but also provide a reliable diagnosis of pathological conditions. 

To date, a variety of sensors have been developed based on different analytical techniques to detect H_2_O_2_ in the cellular environment [30,31,32,33,34,35]. Among them, the electrochemical method offers the advantages of low cost, fast response, high sensitivity, simple instrumentation, and label-free detection [36,37,38,39,40,41,42,43]. Many electrochemical sensors have been developed for direct and real-time detection of cellular H_2_O_2_ using different electrodes based on emerging materials, such as graphene [40,43,44], Au–TiO_2_ [38], MoS_2_ nanoparticles [41], Mn_2_CuO_4_ microspheres [36], ZnMn_2_O_4_@rGO/GCE [39], GO-AuNP/ITO [37], etc. However, most sensors are made with firm and rigid electrodes, which do not effectively comply with elastic and curved cells. Since mechanical stress caused by the contact between cells and the substrate could influence the H_2_O_2_ expression of the cells [45,46,47], flexible electrodes are suitable substrates for enhanced biocompatibility, improved cell adhesion, increased stability during cell culture, and monitoring of H_2_O_2_ released from the cells.

SWCNTs offer several advantages for biosensors, including high electronic conductivity, minimal surface fouling, chemical stability, and high sensitivity due to their one-dimensional quantum confinement properties [48,49]. Moreover, SWCNTs integrated into PET (SWCNT/PET) offer flexibility, transparency, and transducing capability, making them attractive for wearable biosensors [50,51]. However, SWCNT/PET electrodes lack catalytic activity toward H_2_O_2_.

**Scheme 1:**
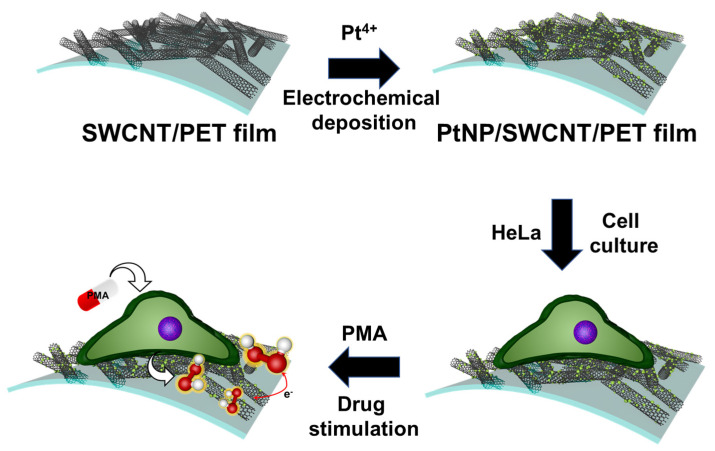
Fabrication of a flexible electrochemical sensor based on SWCNT/PET film functionalized with PtNP and its sensing principle for real−time monitoring of H_2_O_2_ release from living cells.

To address this issue, we functionalized a SWCNT/PET film electrode with platinum nanoparticles (PtNPs) to develop a nonenzymatic, flexible electrochemical sensor for in situ monitoring of H_2_O_2_ (Scheme 1). The PtNPs improve the conductivity and biocompatibility of the film, while their inherent electrocatalytic activity toward H_2_O_2_ enables the sensitive and selective detection of H_2_O_2_ [52,53,54]. The SWCNT network also acts as a stable conductive substrate that maintains stability under mechanical stress. This substrate also offers several advantages over traditional rigid substrates, including improved flexibility, biocompatibility, and transparency. These features enable real-time monitoring of H_2_O_2_ release from living cells, which is critical for understanding the dynamics of cellular processes. 

Overall, flexible substrates represent a significant advancement in the field of electrochemical sensing, particularly for applications involving living cells. Our work demonstrates the potential of flexible substrates for real-time monitoring of H_2_O_2_ release from living cells and highlights the importance of using appropriate substrates for successful electrochemical sensing in biological systems.

## 2. Materials and Methods

### 2.1. Materials

Chloroplatinic acid hexahydrate (H_2_PtCl_6_•6H_2_O), potassium ferricyanide (K_3_Fe(CN)_6_), sulfuric acid (H_2_SO_4_), phorbol 12-myristate 13-acetate (PMA), glucose, cysteine, 4-acetamidophenol, glutamic acid, dopamine (DA), L-(+)-ascorbic acid (AA), uric acid (UA), acetone, methanol, and phosphate-buffered saline (PBS) were purchased from Sigma-Aldrich. Hydrogen peroxide (H_2_O_2_) was obtained from Samchun pure chemical co, LTD, Pyeongtaek, Korea. H_2_O_2_ was prepared in 10 mM phosphate-buffered saline (PBS) solutions at pH 7.4. All chemicals used in this study were of analytical grades, and aqueous solutions were prepared with deionized (DI) water. DI water was prepared using water purification systems (specific resistivity >18 MΩ cm, Milli-Q, Millipore Korea, Co., Ltd., aqua Max Ultra 370, Younglin Instrument Co., Anyang, Gyounggi-do, Korea). Hoechst 33342 Trihydrochloride trihydrate (Hoechst 33342) was obtained from Thermo Fisher Scientific. AZ 5214-E photoresist (PR) and AZ 300 MIF Developer were purchased from AZ Electronic Materials (Luxembourg, UK).

### 2.2. Instrumentation

All electrochemical measurements were performed using a CHI 760E electrochemical workstation (CH Instruments, Inc., Bee Cave, TX, USA). The working electrode used was a flexible SWCNT/PET film, which was prepared by coating SWCNT onto a flexible poly(ethylene terephthalate) (PET) substrate using a vacuum filtration method (Topnanosys Co., Cheonan, Chungcheongnam-do, Republic of Korea). A platinum wire and an Ag/AgCl electrode (saturated KCl) were employed as the counter electrode and reference electrode, respectively. Contact angle measurements were performed on a PHOENIX-MINI (SEO Co, Ltd., Suwon, Gyeonggi-do, Republic of Korea). FE-SEM images and the EDX spectrum were taken on a SIGMA500 (Carl ZEISS, Jena, Germany 1846). The biocompatibility images were obtained by an FV-10i confocal microscope (Olympus Co., Ltd., Shinjuku-ku, Tokyo, Japan). The optical transmittances of SWCNT network films were recorded by an ultraviolet-visible near-infrared (UV–vis-NIR) spectrophotometer V-670 (Jasco International Co., Ltd., Hachioji City, Tokyo, Japan). 

### 2.3. Fabrication of PtNP/SWCNT/PET Network Film Electrode

The fabrication of PtNPs was carried out using a similar method as in our previous studies [55]. To synthesize and deposit PtNPs on the SWCNT film, the film was immersed in DI water containing 1.0 mg/mL Pt^4+^ ion. Electrodeposition of Pt was performed by cyclic voltammetry (CV) technique at a scan rate of 50 mV/s for 20 cycles, using a potential range of −0.2 V to 0.8 V. After being rinsed with DI water and dried with N_2_ gas, the PtNP/SWCNT network film electrode was obtained. The geometrical surface area of the working electrode was 0.0706 cm^2^. For a comparative study to monitor H_2_O_2_ release from cells in real time, interdigitated array electrodes (IDEs) with different pairs of PtNP/SWCNT and/or bare SWCNT electrodes were fabricated. The IDE was composed of 16 electrode fingers with a width of 100 μm, a gap of 100 μm, and a length of 1.95 mm. The IDE was patterned on the SWCNT/PET film using the standard photolithography method. The SWCNT film was first deposited with AZ 5214-E PR through a spin-coating process at 3000 rpm for 30 s, resulting in a film thickness of 1.62 μm. The film was then soft-baked at 80 °C for 1 min to remove moisture on the surface of the substrate and the standing wave on the positive PR layer. Next, UV light exposure was conducted for 30 s to pattern-transfer the IDE mask on the SWCNT/PET film surface. For the development process, AZ 300 MIF developer was used twice at room temperature for 10 min. Finally, an etching process using O_2_ plasma for 20 min was carried out to remove an unexposed area.

### 2.4. Cell Culture and Imaging

The human cervical cancer cell line HeLa was obtained from the Korean cell line bank (KCLB, Seoul, Korea). Cells were grown in Roswell Park Memorial Institute (RPMI) 1640 medium (Cellgro, Manassas, VA, USA) supplemented with 10% fetal bovine serum (FBS; Equitech-bio, Kerrville, TX, USA) and 1% penicillin–streptomycin (APS) at 37 °C in a humidified atmosphere containing 5% CO_2_. To test the cell viability, the PtNP/SWCNT network film (1.5 cm × 3 cm) was prepared and sterilized by irradiating it with UV for 2 h before seeding the cells. Then, HeLa cells at a density of ~5 × 10^4^ cell cm^−2^ were seeded on the PtNP/SWCNT network film electrode. After 24 h, 48 h, and 72 h of incubation, the cells were stained with Hoechst 33342 for fluorescence imaging to compare the biocompatibility.

## 3. Results

### 3.1. Preparation and Characterization of PtNP/SWCNT Network Film

**Figure 1:**
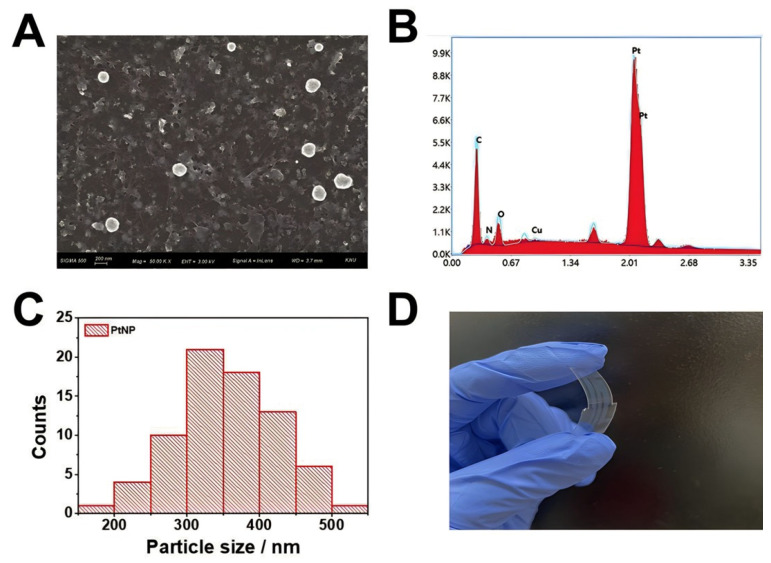
(**A**) FE-SEM image and (**B**) EDX spectrum of PtNP/SWCNT network film. (**C**) Histogram of particle size distribution of PtNP. (**D**) Photograph of bent PtNP/SWCNT network film.

A transparent and flexible SWCNT network film was functionalized with PtNPs via electrochemical deposition with a potential range of −0.2 V to 0.8 V in 1 mg/mL Pt^4+^ in 10 mM PBS (pH 7.4) for 20 cycles using the CV method. As shown in Figure S1 (Supplementary Material), the oxidation and reduction currents gradually increased with each scan cycle. After the redox process with multiple CV scans, PtNPs were successfully formed on the SWCNT network film, while the SWCNTs remained well-distributed and networked with each other on a PET film without any discernible morphological change in the FE-SEM images (Figure 1A). To confirm the presence of PtNPs on the SWCNT surface, the elemental compositions were analyzed using EDS (Figure 1B). The EDS spectrum showed characteristic signals of C, O, and Pt, which identified the formation of PtNPs via electrochemical deposition on the SWCNT network film. The corresponding particle size distribution histogram is shown in Figure 1C; the measured size of PtNPs was 360 nm ± 70 nm for the average diameter with standard deviation. Figure 1D displays the flexible system based on PtNP/SWCNT network films, showing high transparency with a transmittance value of around 80% in the Vis-NIR range (Figure S2). As shown in Figure S2, the transmittance did not change significantly after the electrodeposition process of PtNPs on SWCNT network films.

**Figure 2:**
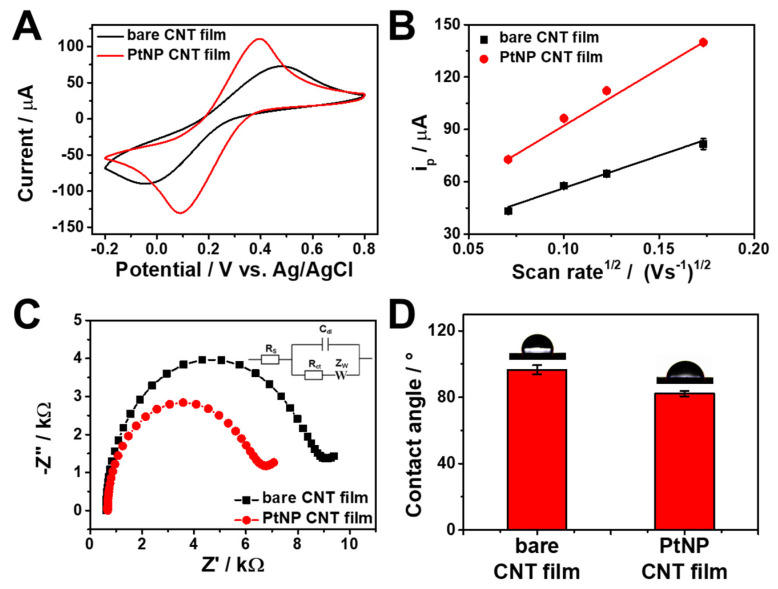
(**A**) CV curves of bare SWCNT and PtNP/SWCNT network film in 0.1 M KCl containing 10 mM [Fe(CN)_6_]^3−^ at a scan rate of 15 mV/s. (**B**) The corresponding plots for cathodic peak currents vs. the square root of the scan rate. (**C**) Nyquist plots of bare SWCNT film and PtNP/SWCNT film in 0.1 M KCl containing 10 mM [Fe(CN)_6_]^3−^. Inset: a Randles equivalent circuit diagram. (**D**) Contact angle measurements. Images of a water droplet on the surfaces of bare SWCNT film and PtNP/SWCNT film.

The electron transfer kinetics of the PtNP/SWCNT network film were studied by voltammetric and impedimetric measurements utilizing [Fe(CN)_6_]^3−^ as a redox probe. Figure 2A shows the voltammetric responses of bare SWCNT and PtNP/SWCNT films in 0.1 M KCl containing 10 mM [Fe(CN)_6_]^3−^. The bare SWCNT film exhibited a pair of redox peaks with peak-to-peak separation (ΔE_p_) of 0.62 V, which suggests an irreversible process attributable to slow diffusion through the narrow pores and sluggish electron transfer at the SWCNT surface caused by a graphite basal-plane-like structure [56]. On the contrary, PtNP/SWCNT network film showed better electrochemical performance (quasireversible) with a smaller ΔE_p_ of 0.26 V and enhanced redox peak currents (i_p_) owing to a faster electron transfer on PtNPs and the large effective surface area of the PtNP/SWCNT film. The effective surface area of the PtNP/SWCNT network film was determined from CV curves obtained from 0.1 M KCl containing 10 mM [Fe(CN)_6_]^3−^ at varying scan rates (Figure S3). The corresponding plot (Figure 2B) shows a linear relation between i_p_ and the root-squared scan rate, which is consistent with the Randles–Sevick model (under nonstandard conditions) for diffusion-controlled transport of species [57,58,59,60]. The determined surface area of the PtNP/SWCNT film was 0.00578 cm^2^, which is larger than that of bare SWCNT film (0.00409 cm^2^).

(1) $$I_{pf}^{rev}=\pm 0.446nFAC\sqrt{\frac{nFDv}{RT}}$$

(2) $$I_{pf}^{quasi}=\pm 0.436nFAC\sqrt{\frac{nFDv}{RT}}$$

(3) $$I_{pf}^{irrev}=\pm 0.496\sqrt{\alpha n^{\prime }}nFAC\sqrt{\frac{nFDv}{RT}}$$

EIS measurements also confirm the formation of PtNP on the SWCNT surface and a faster electron transfer on the PtNP/SWCNT network film compared with the bare SWCNT film (Figure 2C). The Nyquist plots demonstrate a significant drop in the diameter of the semicircle after the electrodeposition of PtNP on the SWCNT surface, indicating a decrease in the charge transfer resistance (R_ct_) and an enhanced electron transfer of the redox probe at the PtNP/SWCNT surface. The values of R_ct_ calculated from a semicircle in the high-frequency region of the Nyquist plot were 5.47 kΩ and 7.75 kΩ for PtNP/SWCNT and bare SWCNT films, respectively. The lower impedance interface of the PtNP/SWCNT films was mainly attributed to the higher conductivity of the electrodeposited platinum. Since cell adhesion and interaction on the electrode substrate depends on the hydrophilicity of the surface of the electrode, the wetting contact angle of the PtNP/SWCNT films was investigated. The wetting characteristics of bare SWCNT and PtNP/SWCNT network films are shown in Figure 2D, where the average equilibrium static contact angles were found to be 97° and 82°, respectively. The electrodeposition of PtNPs on the SWCNT film resulted in a more hydrophilic surface, which may be attributed to the hydrophilic property of PtNPs [61], as well as the enhanced roughness of the film surface.

### 3.2. Electrochemical Sensing Performance toward H_2_O_2_

**Figure 3:**
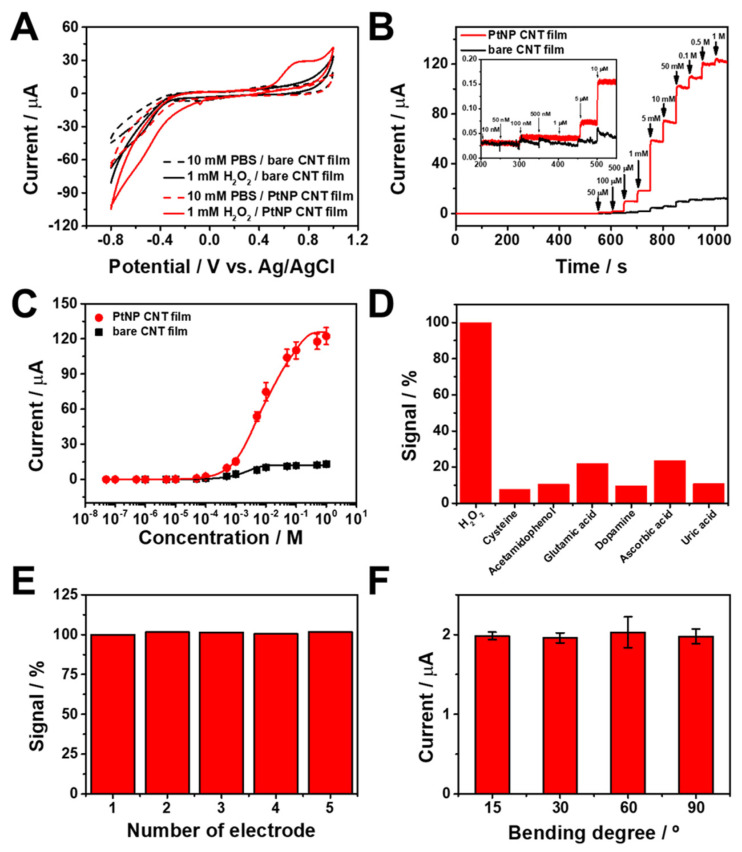
(**A**) CV curves of bare SWCNT (black line) and PtNP/SWCNT (red line) network films in 10 mM PBS (pH 7.4) in the absence (dashed line) and the presence (solid line) of 1 mM H_2_O_2_ at a scan rate of 50 mV/s. (**B**) Amperometric responses of bare SWCNT (black line) and PtNP/SWCNT (red line) network films to successive addition of H_2_O_2_ to PBS (10 mM, pH 7.4) at a fixed potential of 0.6 V under stirring and (**C**) the corresponding calibration curves. (**D**) Selectivity of PtNP/SWCNT network film sensor. Signals were from each amperometric current of 10 μM H_2_O_2_, 100 μM cysteine, 4−acetamidopenol, glutamic acid, dopamine, ascorbic acid, and uric acid were compared to to PBS (10 mM, pH 7.4) at a fixed potential of 0.6 V under stirring. (**E**) Sensor reproducibility for five different modified electrodes. Signals were from each i_p_ in the CV curves of 1 mM H_2_O_2_ at 0.6 V were compared. (**F**) Effects of different bending angles (15, 30, 60, and 90°) on the current response to 100 μM H_2_O_2_ using flexible PtNP/SWCNT film.

In order to investigate the impact of Pt on the sensor’s performance, we compared the electrochemical catalytic behavior of the PtNP/SWCNT network film with that of the bare SWCNT film using CV. Figure 3A shows the voltammetric responses toward 1 mM of H_2_O_2_ in 10 mM PBS at pH 7.4. No noticeable signal was detected in the absence of H_2_O_2_ for either bare SWCNT or PtNP/SWCNT network films. However, the addition of 1 mM H_2_O_2_ resulted in significant redox currents originating from the catalytic oxidation and reduction of H_2_O_2_ at the surface of the electrode. Notably, the current response of the PtNP/SWCNT network film was much higher than that of the bare SWCNT. This improvement in electrocatalytic activity is attributed to the higher electrocatalytic performance and good conductivity of the PtNPs deposited on the SWCNT films. These results demonstrate the potential of flexible and transparent PtNP/SWCNT network film for the detection of H_2_O_2_. It is worth noting that the electrodeposition of PtNPs on the SWCNT surface was further confirmed by the altered electrocatalytic activity of the SWCNT film for H_2_O_2_ redox processes.

To assess the analytical performance of the PtNP/SWCNT network film, we conducted chronoamperometric measurements in 10 mM PBS. The applied working potential was 0.6 V, at which H_2_O_2_ oxidizes. The oxidation of H_2_O_2_ is more sensitive and reproducible compared to its reduction, since oxygen does not contribute to the background current [62,63]. On the other hand, the reduction of H_2_O_2_ at a negative potential may not easily produce a reproducible amperometric signal, since the H_2_O_2_ signal would be obscured by the oxygen reduction [62]. Figure 3B displays the typical amperometric responses of the PtNP/SWCNT network film alongside the bare SWCNT film upon adding H_2_O_2_ at a selected working potential of 0.6 V. The PtNP/SWCNT network film electrode exhibits highly sensitive amperometric responses compared to the bare SWCNT film. The electrode showed a quick current response that could achieve a dynamic equilibrium of current signal under 5 s after adding H_2_O_2_, reflecting a favorable electron transfer between PtNP/SWCNT network film and H_2_O_2_. H_2_O_2_ began to trigger a response from the PtNP/SWCNT network film at a concentration of 500 nM, and subsequent additions of H_2_O_2_ resulted in proportional increases in current. Figure 3C shows the calibration curve for H_2_O_2_ measurements with a PtNP/SWCNT network film sensor, demonstrating a wide dynamic range (500 nM-1 M) in which there were detectable signals and a highly sensitive linear range with a steep slope of approximately 44 per decade. For the linear range, the regression equation is represented as i (µA) = 156 + 44.2 log [H_2_O_2_] (M) (correlation coefficient, R^2^ = 0.982). In the case of bare SWCNT film, the dynamic range is 1 μM to 1 M, and the regression equation for the linear range is represented as i (µA) = 11.4 + 2.59 log [H_2_O_2_] (M) (correlation coefficient, R^2^ = 0.980). The limit of detection (LOD) of the sensor for H_2_O_2_ was estimated to be 228 nM at a signal-to-noise (S/N) ratio of 3, demonstrating superior electrochemical sensing ability compared to the bare SWCNT film (LOD = 14.0 μM). Table 1 shows a comparison the H_2_O_2_ detection performance of our electrochemical biosensors with that of other existing biosensors. As demonstrated in Table 1, the PtNP/SWCNT network films exhibit superior analytical performance for H_2_O_2_ detection in comparison to other electrochemical biosensors. The flexible electrode exhibits a relatively lower detection limit. To assess the selectivity of the sensor, we tested its amperometric responses upon the addition of 10 μM of H_2_O_2_ and several other biochemical species, including 100 μM cysteine, 4-acetamidophenol, glutamic acid, dopamine, ascorbic acid, and uric acid. As shown in Figure 3D, only the introduction of H_2_O_2_ resulted in a noticeable change in the amperometric response, while other biochemical species, even at concentrations ten times higher, produced no discernible effect, indicating excellent selectivity of the sensor. We also evaluated the reproducibility of the fabricated sensors by recording CV curves of 1 mM H_2_O_2_ on five different PtNP/SWCNT network electrodes prepared under identical conditions. As depicted in Figure 3E, the anodic peak currents at 0.6 V showed an average relative standard deviation (RSD) of only 0.7%, demonstrating the sensors’ reproducibility. We also evaluated the stability of the PtNP/SWCNT network film in a cell culture medium without cells (Figure S5). The signal was assessed by comparing the amperometric current of 100 μM H_2_O_2_ in 10 mM PBS. After 120 h, the PtNP/SWCNT network film retained 95.1% of its initial response, with an RSD value of 8.0%, indicating excellent stability of the sensor. Furthermore, we examined the effects of bending stress on the overall sensing performance of the PtNP/SWCNT network film, as flexibility is critical for practical biosensor devices. The sensor showed negligible changes when bent inward at angles of 15°, 30°, 60°, and 90°, with an average RSD of 1.3% in the amperometric response towards 100 μM H_2_O_2_. The signals were measured in triplicate (n = 3). The sensor’s stability under bending cycles was also evaluated using a homemade bending apparatus. The bending cycle stability of the PtNP/SWCNT network film electrode was evaluated up to 500 bending cycles at 60° (Figure S4), demonstrating excellent stability, with approximately 94% signal retention after 300 cycles and 81% signal retention after 500 cycles of bending, with an average RSD of 1.29% in the current response towards 100 μM H_2_O_2_.

### 3.3. Real-Time Monitoring of H_2_O_2_ Release from Living Cells

**Figure 4:**
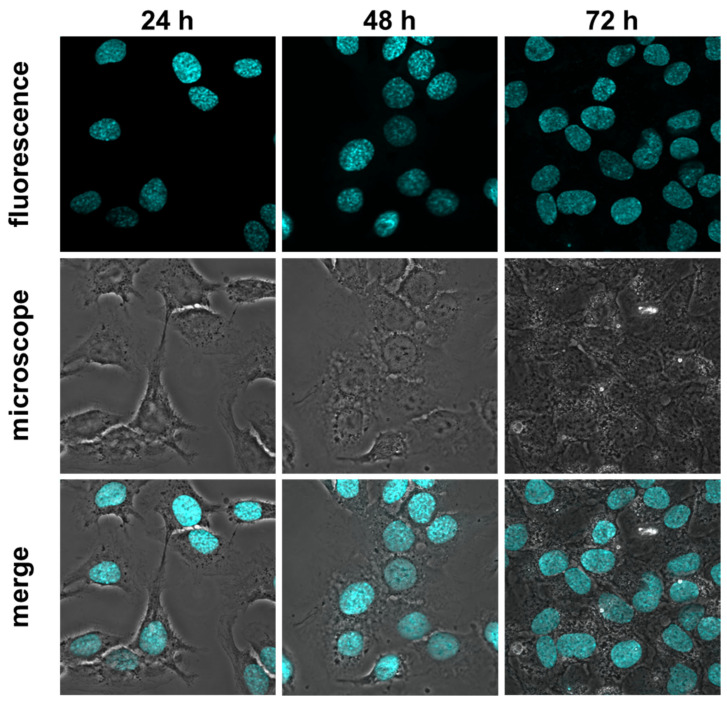
Fluorescence and microscopic for the biocompatibility test of HeLa cells cultured on PtNP/SWCNT network film for 24 h, 48 h, and 72 h. Cyan color represents Hoechst 33342-stained nuclei observed at 440–480 nm with 358 nm excitation.

HeLa cells were cultured on the surface of the sterilized PtNP/SWCNT network film to show the viability of the PtNP/SWCNT network film as a flexible sensor for the in situ monitoring of cell exocytosis. The sensor’s biocompatibility was first examined by fluorescence and microscope images. Figure 4 shows the images of HeLa cells seeded on the sensor for 24 h, 48 h, and 72 h cultivation. After 24 h, HeLa cells on the sensor formed pseudopodia and were firmly adhered to the surface. After 48 h of culture, the cells grew and proliferated well on the surface, with most cells exhibiting a typical spindle structure. As the culture progressed to 72 h, HeLa cells with a spindle shape continued to proliferate effectively and nearly cover the whole surface. Fluorescence images clearly showed that most cells stained by Hoechst 33342 (cyan) were alive on the surface, demonstrating the sensor’s outstanding biocompatibility for the adhesion and growth of mammalian cells.

**Figure 5:**
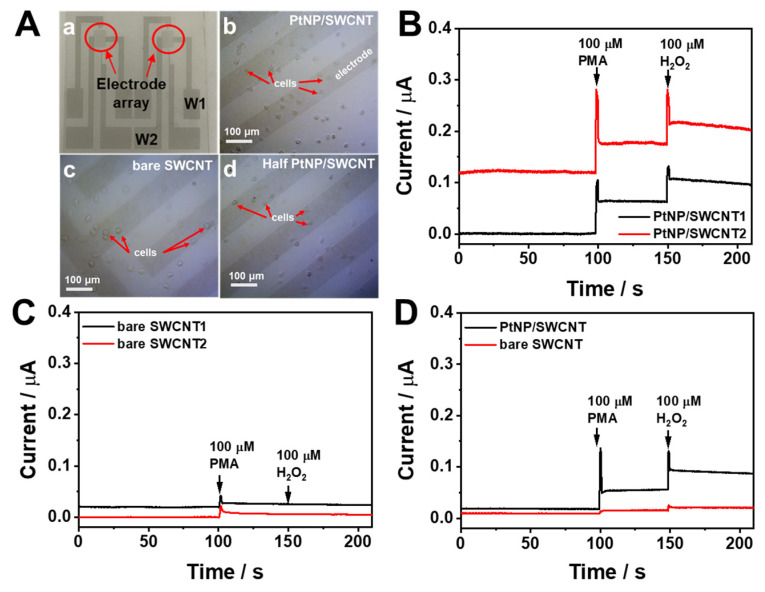
(**A**) (**a**) Photograph of IDEs and magnified images of IDE patterns with (**b**) pairs of PtNP/SWCNTs, (**c**) pairs of bare SWCNTs, and (**d**) pairs of half bare SWCNT and half PtNP/SWCNT cultured with HeLa cells. Monitoring of H_2_O_2_ release from HeLa cells using IDEs with (**B**) pattern (**b**), (**C**) pattern (**c**), and (**D**) pattern (**d**) upon the addition of 100 μM PMA at 0.6 V in 10 mM PBS (pH 7.4).

Along with advantageous properties including flexibility, transparency, large surface area, good biocompatibility, and high conductivity, the PtNP/SWCNT film can be patterned into various shapes. Therefore, we prepared IDE sensors with three different electrode patterns using a standard photolithography method: pairs of PtNP/SWCNTs, pairs of bare SWCNTs, and pairs of half bare SWCNT and half PtNP/SWCNT. These sensors were used to monitor H_2_O_2_ release from HeLa cells cultured on the electrode surface by the amperometric method. Figure 5A displays the fabricated IDEs with different electrode pairs of PtNP/SWCNT and bare SWCNT. For in situ monitoring of H_2_O_2_ released from live cells, HeLa cells were cultured on the fabricated IDEs (Figure 5A(b–d)). The electrochemical system was set up using an IDE as the working electrode, as depicted in Figure S6. In this configuration, a platinum wire was utilized as the counter electrode, and an Ag/AgCl electrode with saturated KCl served as the reference electrode (Figure S6A). Additionally, we employed a specially designed electrochemical cell with an active site diameter of 3 mm, as illustrated in Figure S6B. The generation of H_2_O_2_ from HeLa cells was induced by adding PMA as a common stimulant, which activates a series of signaling pathways, including the oxidation of O_2_, the generation of O_2_^−^, and release of H_2_O_2_ to the extracellular environment [68,69]. Figure 5B shows a sharp increase in current on the IDE with pairs of PtNP/SWCNTs upon the addition of 100 μM PMA. To confirm that this current increase was caused by H_2_O_2_, 100 μM H_2_O_2_ was added, which led to a similar current increase, suggesting that the current response was due to the oxidation of H_2_O_2_ released from the HeLa cells cultured therein. In contrast, the IDE with pairs of bare SWCNTs exhibited a negligible current increase after the addition of the same amount of PMA and even H_2_O_2_ (Figure 5C), indicating the significance and electrocatalytic effect of PtNPs on the SWCNT film. In the case of the IDE with pairs of half PtNP/SWCNT and half bare SWCNT, upon the stimulation of PMA, the HeLa cells immediately produced a distinct amperometric signal only on the PtNP/SWCNT pattern (black line in Figure 5D). On the bare SWCNT pattern, the measured response was insignificant (red line in Figure 5D), reflecting the superior sensing capability of PtNP/SWCNT network film for real-time monitoring of H_2_O_2_ released from the cells. In contrast, when the amperometric response was measured in the absence of HeLa cells using the PtNP/SWCNT film (Figure S7), there was no noticeable change observed following the addition of 100 μM of PMA, unlike the significant signal observed with 100 μM of H_2_O_2_.

**Figure 6:**
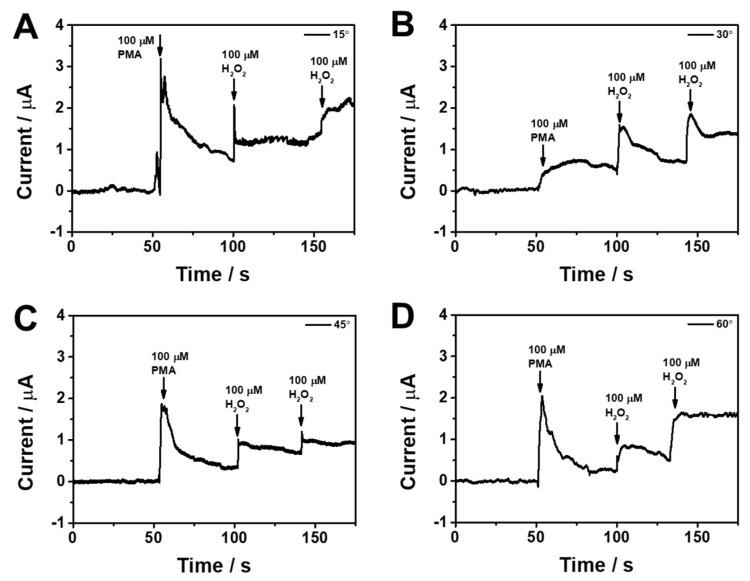
Monitoring of H_2_O_2_ release from HeLa cells using the PtNP/SWCNT network film sensor upon the addition of 100 μM PMA at 0.6 V in 10 mM PBS (pH 7.4) under different bending states at angles of (**A**) 15°, (**B**) 30°, (**C**) 45°, and (**D**) 60°.

To evaluate the flexibility of the PtNP/SWCNT film sensor in detecting biochemical signals from mechanically deformed cells, we tested its performance in real-time and in situ monitoring of H_2_O_2_ even when bent. Different mechanical bending strains were applied to the sensor with HeLa cells cultured on it. Figure 6 shows the amperometric responses of cell-cultured PtNP/SWCNT film sensors under different bending states at angles of 15°, 30°, 45°, and 60° upon the addition of 100 μM PMA and H_2_O_2_ at 0.6 V in 10 mM PBS (pH 7.4). The average signals measured from 100 μM PMA and H_2_O_2_ at all bending states were calculated as 0.99 μM and 0.56 μM, respectively. The corresponding relative standard deviation (RSD) values were calculated as 10.7% and 7.1%, respectively. These results indicate that the signal varies with the bending angle, as evidenced by the calculated RSD values. However, considering the intended application of the biosensor for a wearable device, these variations are still considered feasible. PMA stimulation still caused a significant current increase on all sensors under different bending states (Figure 6A–D), which is attributed to the H_2_O_2_ secreted from the cells. This indicates that the cells at the deformed states could secrete H_2_O_2_. In addition, a staircase increase in current was observed upon the following successive addition of H_2_O_2_, suggesting that mechanical deformation has little effect on real-time and in situ monitoring of H_2_O_2_.

## 4. Conclusions

Real-time and in situ monitoring of H_2_O_2_ release from living cells was successfully achieved using a flexible and transparent PtNP/SWCNT network electrode. The electrode was fabricated by the electrodeposition of PtNPs onto SWCNT/PET film after multiple CV cycles. The resulting electrode demonstrated remarkable mechanical, physicochemical, and electrochemical properties due to the synergistic impact of different components in the PtNP/SWCNT network film, which led to enhanced nonenzymatic H_2_O_2_ detection. The electrode also showed good biocompatibility, enabling conformal cell growth on its surface with excellent adhesion. These features allowed for in situ tracking of H_2_O_2_ secreted from live cells under steady or mechanically deformed states. This approach offers a promising strategy to develop flexible and transparent sensors for real-time and in situ monitoring of electroactive species related to cell metabolism and for various biomedical applications. Moreover, it could serve as a potent tool for the development of wearable biosensing devices in the future.

## Figures and Tables

**Table 1:** Comparison of nonenzymatic electrochemical biosensors for H_2_O_2_ detection.

Electrode	Substate	Linear Range (μM)	Detection Limit (μM)	Ref.
Graphitic carbon nitride/Poly(3-hexylthiophene)	Rigid	1–800	0.38	[64]
Pt-Pd nanocoral/carbon fiber microelectrode	Rigid	5–3920	0.42	[65]
Pt nanoparticles/multiwalled carbon nanotubes/CPE	Flexible	0.5–100	0.2	[66]
Au nanoflower-modified IL functionalized graphene framework	Flexible	410–3300	0.1	[67]
PtNP/SWCNT network film	Flexible	0.5–1,000,000	0.228	This work

## Data Availability

The data that support the findings of this study are available from the corresponding author upon reasonable request.

## Supplementary Materials

The following supporting information can be downloaded at: https://www.mdpi.com/article/10.3390/bios13070704/s1, Figure S1: CV curves of SWCNT/PET film electrode in 1 mg/mL chloroplatinic acid hexahydrate at a scan rate of 50 mV for 20 cycles; Figure S2: Transmittance of bare SWCNT, flat PtNP/SWCNT film, and bent PtNP/SWCNT film; Figure S3: CV curve of (A) bare SWCNT and (B) PtNP/SWCNT film in 0.1 M KCl containing 10 mM [Fe(CN)_6_]^3−^ at a various scan rates; Figure S4: Effects of bending cycles on the current response to 100 μM H_2_O_2_ using PtNP/SWCNT film bending at 60°; Figure S5: Stability of PtNP/SWCNT network film sensor in cell culture media without cells. Signals from each amperometric response of 100 μM H_2_O_2_ in 10 mM PBS were compared; Figure S6: (A) Three-electrode setup including the IDE as working electrodes. (B) Expanded view of the electrochemical cell; Figure S7: Monitoring of 100 μM PMA and 100 μM H_2_O_2_ using the flexible PtNP/SWCNT film without HeLa cells.
