# Supplementary material for: A Flexible and Transparent PtNP/SWCNT/PET Electrochemical Sensor for Nonenzymatic Detection of Hydrogen Peroxide Released from Living Cells with Real-Time Monitoring Capability
Source: Biosensors (Basel). 2023 Jul 3;13(7):704. doi: 10.3390/bios13070704 (PMC10377607; doi:10.3390/bios13070704)
Supplement: Supplementary file 1 [file biosensors-13-00704-s001.zip › biosensors-2462130-supplementary.pdf]

Supplementary Materials

A Flexible and Transparent PtNP/SWCNT/PET Electrochemical Sensor for Nonenzymatic Detection of Hydrogen Peroxide Released from Living Cells with Real-Time Monitoring Capability

Da Eun Oh [1,†], Chang-Seuk Lee [2,†], Tae Wan Kim [3], Seob Jeon [4] and Tae Hyun Kim [1,*]

1 Department of Chemistry, Soonchunhyang University, Asan 31538, Republic of Korea
2 Department of Chemistry, Seoul Woman's University, Seoul 01797, Republic of Korea
3 Department of Medical Life Science, Soonchunhyang University, Asan 31538, Republic of Korea
4 Department of Obstetrics and Gynecology, College of Medicine, Soonchunhyang University Cheonan Hospital, Cheonan 31151, Republic of Korea
* Correspondence: thkim@sch.ac.kr
† These authors contributed equally to this work.

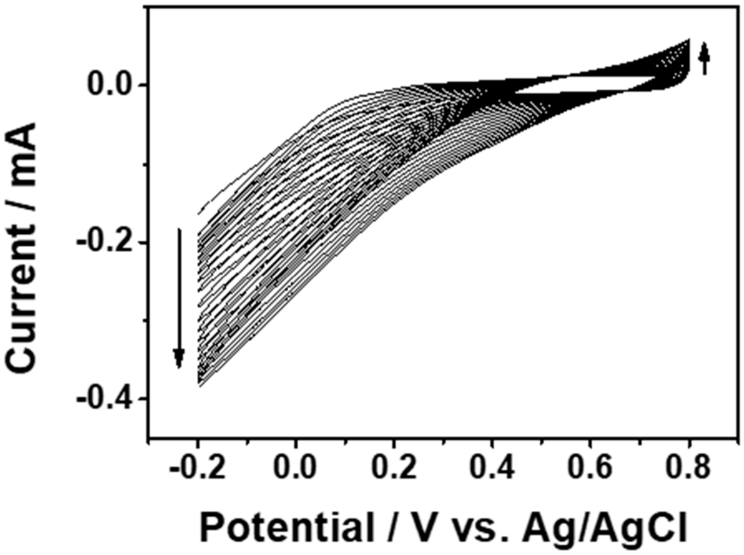

Figure S1. CV curves of SWCNT/PET film electrode in 1 mg/mL chloroplatinic acid hexahydrate at a scan rate of 50 mV for 20 cycles.

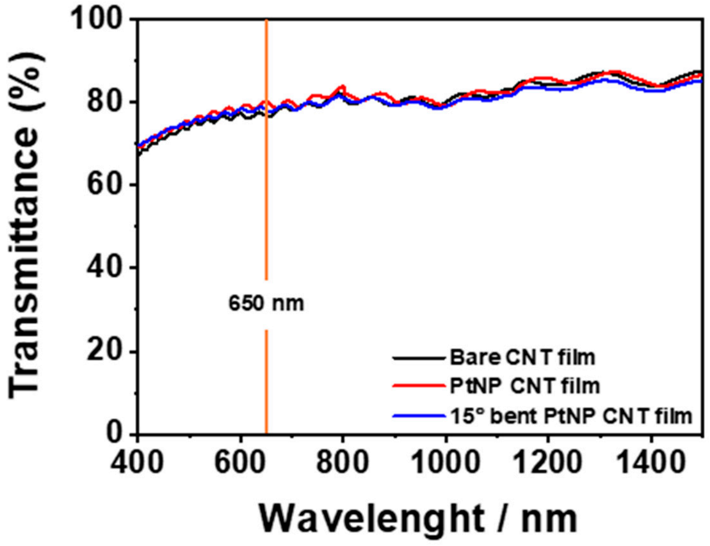

Figure S2. Transmittance of bare SWCNT, flat PtNP/SWCNT film, and bent PtNP/SWCNT film.

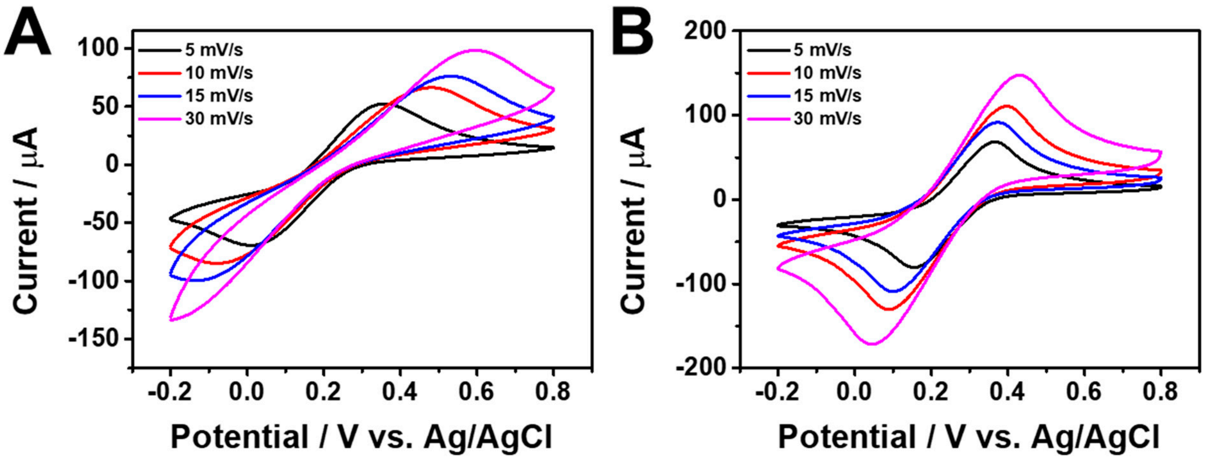

Figure S3. CV curve of (A) bare SWCNT and (B) PtNP/SWCNT film in 0.1 M KCl containing 10 mM $[Fe(CN)_6]^{3-}$ at a various scan rate.

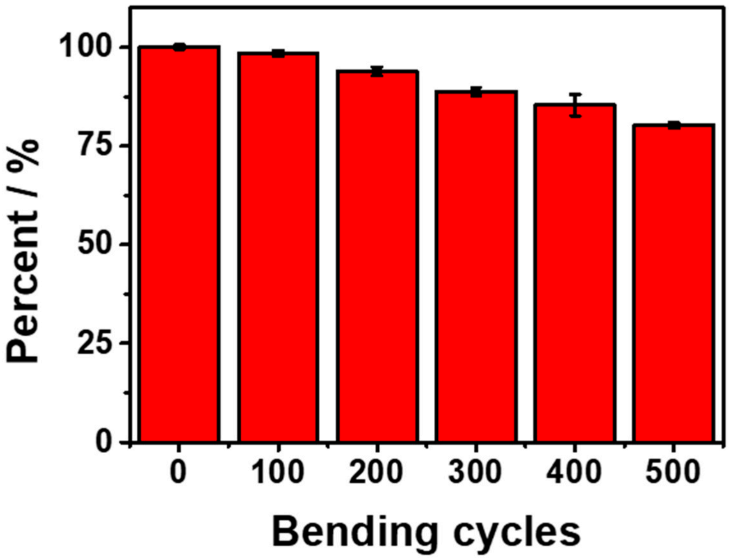

Figure S4. Effects of bending cycles on the current response to 100 μM H_2O_2 using PtNP/SWCNT film bending at 60°.

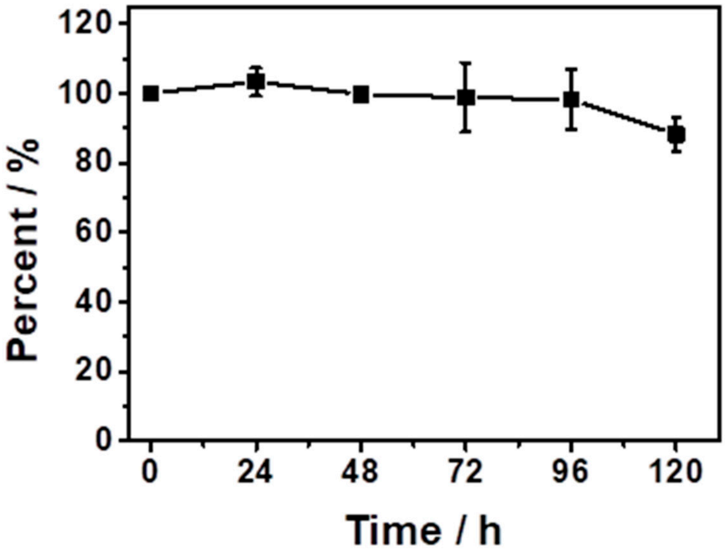

Figure S5. Stability of PtNP/SWCNT network film sensor in cell culture media without cells. Signals were compared from each amperometric response of 100 μM H_2O_2 in 10 mM PBS.

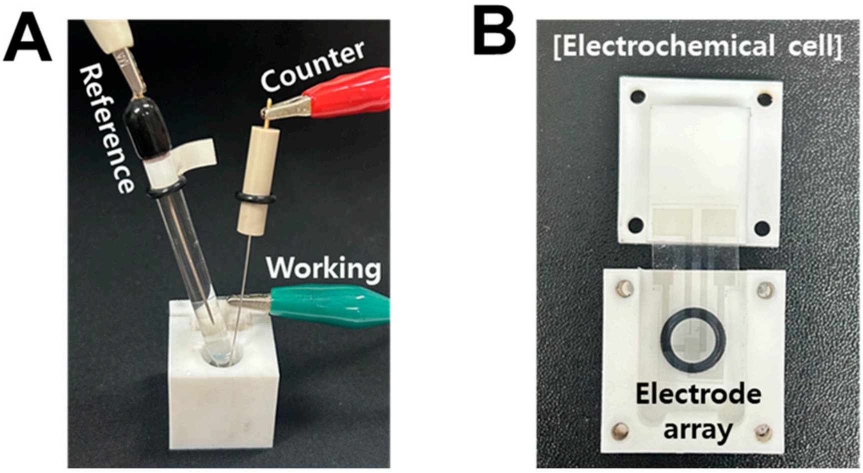

Figure S6. (A) Three-electrode setup includes the IDE as working electrodes. (B) Exploded view of the electrochemical cell.

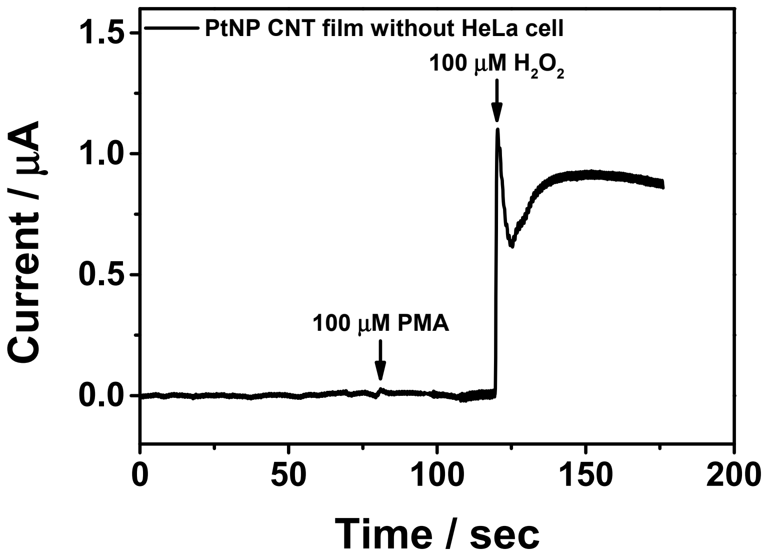

Figure S7. Monitoring of 100 μM PMA and 100 μM H_2O_2 using the flexible PtNP/SWCNT film without HeLa cell.
